# Cost-Effectiveness of Vaccination of Older Adults with an MF59^®^-Adjuvanted Quadrivalent Influenza Vaccine Compared to Standard-Dose and High-Dose Vaccines in Denmark, Norway, and Sweden

**DOI:** 10.3390/vaccines11040753

**Published:** 2023-03-29

**Authors:** Jorge Jacob, Tor Biering-Sørensen, Lars Holger Ehlers, Christina H. Edwards, Kristin Greve-Isdahl Mohn, Anna Nilsson, Jonas Hjelmgren, Wenkang Ma, Yuvraj Sharma, Emanuele Ciglia, Joaquin Mould-Quevedo

**Affiliations:** 1 IQVIA, London W2 1AF, UK; 2 Department of Cardiology, Herlev and Gentofte Hospital, 2730 Herlev, Denmark; 3 Center for Translational Cardiology and Pragmatic Randomized Trials, Department of Biomedical Sciences, Faculty of Health and Medical Sciences, University of Copenhagen, 2200 Copenhagen, Denmark; 4 Nordic Institute of Health Economics, 8000 Aarhus, Denmark; 5 Department of Public Health and Nursing, Norwegian University of Science and Technology, 7491 Trondheim, Norway; 6 Influenza Centre, Department of Clinical Science, University of Bergen, 5020 Bergen, Norway; 7 Department of Medicine, Haukeland University Hospital, 5021 Bergen, Norway; 8 Infectious Disease Unit, Malmö, Skåne University Hospital, 214 28 Malmö, Sweden; 9 The Swedish Institute for Health Economics, 223 61 Lund, Sweden; 10 CSL Seqirus, 81929 Munich, Germany; 11 CSL Seqirus, Summit, NJ 07901, USA

**Keywords:** cost-effectiveness, seasonal influenza, adjuvanted influenza vaccine, Nordic countries

## Abstract

Individuals aged 65 years and above are at increased risk of complications and death from influenza compared with any other age group. Enhanced vaccines, as the MF59^®^-adjuvanted quadrivalent influenza vaccine (aQIV) and the high-dose quadrivalent influenza vaccine (HD-QIV), provide increased protection for older adults in comparison to the traditional standard-dose quadrivalent influenza vaccines (SD-QIV). This study aimed to assess the cost-effectiveness of aQIV compared to SD-QIV and HD-QIV in Denmark, Norway, and Sweden for adults aged ≥65 years. A static decision tree model was used to evaluate costs and outcomes of different vaccination strategies from healthcare payer and societal perspectives. This model projects that compared to SD-QIV, vaccination with aQIV could prevent a combined total of 18,772 symptomatic influenza infections, 925 hospitalizations, and 161 deaths in one influenza season across the three countries. From a healthcare payer perspective, the incremental costs per quality adjusted life year (QALY) gained with aQIV versus SD-QIV were EUR 10,170/QALY in Denmark, EUR 12,515/QALY in Norway, and EUR 9894/QALY in Sweden. The aQIV was cost saving compared with HD-QIV. This study found that introducing aQIV to the entire population aged ≥65 years may contribute to reducing the disease and economic burden associated with influenza in these countries.

## 1. Introduction

Seasonal influenza is an acute respiratory infection caused by influenza viruses [1]. Apart from upper respiratory symptoms, the infection can also lead to acute complications and/or worsening of pre-existing medical conditions such as pneumonia, asthma, myocardial infarction, heart failure, stroke, and kidney injury, increasing the risk of hospitalization and death [2]. Based on estimations of the World Health Organization (WHO), influenza has an annual global attack rate of 5–10% in adults leading to 3–5 million cases of severe illness and 290,000–650,000 deaths every year [1,3]. In the European Union/European Economic Area, influenza causes around 50 million symptomatic cases and 15,000–70,000 deaths annually [4]. Due to increased prevalence of comorbidities, older adults tend to have a higher incidence of influenza-related complications (IRCs), resulting in increased morbidity and mortality [5,6]. During the severe influenza season of 2017/18, the all-cause influenza-attributable mortality was estimated to be 25.4 per 100,000 population for all ages and 118.2 per 100,000 for adults aged ≥65 years in Europe [7,8]. Influenza causes a substantial socioeconomic burden resulting from increased healthcare utilization and work absenteeism. A study conducted in 2014 extrapolated that the total direct and indirect cost of influenza ranges from EUR 6 to 14 billion per year in Europe [9].

Vaccination is the most effective measure to prevent influenza. Health agencies in several European countries support free seasonal influenza programs for high-risk populations, including adults aged ≥65 years and above [10]. Quadrivalent influenza vaccines provide protection against four influenza types (A [H1N1], A [H3N2], B [Victoria], and B [Yamagata]) and have been widely used to replace trivalent influenza vaccines (TIVs), which contained antigens of the two influenza A subtypes and only one influenza B lineage [1]. Standard-dose vaccines provide limited protection for adults ≥65 years because of impaired immunogenicity due to aging (i.e., immunosenescence) among this population [1,11,12]. Enhanced influenza vaccines have been developed in recent years to overcome immunosenescence and enhance immune response in older adults, leading to improved vaccine effectiveness (VE); the high-dose vaccine contains a higher antigen dose (60 µg of viral hemagglutinin antigen per strain, compared to 15 µg in SD vaccines) [12]. The adjuvanted vaccine contains MF59^®^, a squalene oil-in-water emulsion that is designed to induce a more robust, durable, and broader immune response [13]. Enhanced vaccines have shown better protection against influenza infection and hospitalization and a tolerability comparable to SD vaccines among adults aged ≥65 years [12,13,14,15]. In a randomized feasibility trial with 12,477 Danish citizens aged 65 to 79 years (2021/22 influenza season) HD-QIV was associated with lower hospitalization and all-cause mortality rates compared with SD-QIV [16].

As a result, enhanced vaccines have been recommended and/or reimbursed for seasonal influenza vaccination programs for this population in multiple countries [17,18,19,20]. In the 2021/22 influenza season, Denmark recommended SD-QIV for people aged ≥65 years and HD-TIV for adults aged ≥85 years and those residing in nursing homes [21]. In Norway, aQIV has been procured for people aged ≥65 years and living in nursing homes for the influenza season 2022/23 [22]. In Sweden, in addition to SD vaccines which are recommended for the population 6 months and older, aQIV is offered for people in special housing for older adults (SÄBO) [23].

There is no published evidence on the cost-effectiveness of aQIV in comparison to SD-QIV or HD-QIV in the Nordic countries. Recently, aQIV was shown to be a cost-saving option compared with HD-QIV and a cost-effective option compared with SD-QIV for adults over 65 years of age in Spain [24,25]. Similarly, in Germany, aQIV was found to be cost-saving and cost-effective compared to HD-QIV and SD-QIV, respectively, for adults aged ≥65 years [26]. aQIV has also been found to be cost-saving compared with HD-QIV for adults over 65 years of age in the United Kingdom (UK) [27]. In Italy, aQIV was determined to be a cost-effective alternative to SD-QIV [28].

Our study aimed to assess the health economic impact of adopting aQIV compared to SD-QIV and HD-QIV in the seasonal influenza vaccination programs for adults aged ≥65 years in Denmark, Norway, and Sweden.

## 2. Materials and Methods

### 2.1. Model Framework

**Figure 1:**
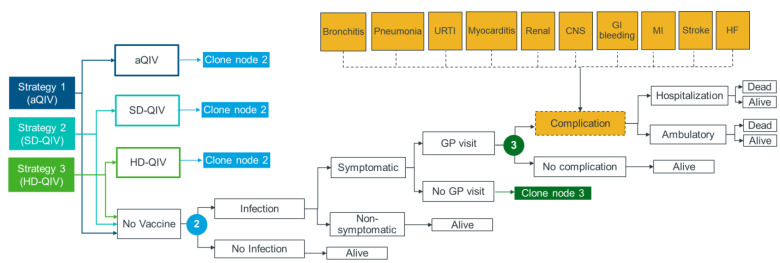
Model structure.

A decision tree model was developed in MS Excel 365 and built on a model structure used in two published cost-effectiveness analyses for influenza vaccines [29,30]. The model (Figure 1) captured key outcomes at different stages of influenza, including infection, developing symptoms, medical attention, developing IRCs, hospitalization, and death.

To allow comparison of different vaccination strategies, the same decision tree pathways starting from the infection stage were replicated for each vaccine (aQIV, SD-QIV, or HD-QIV), allowing outcomes from the populations immunized with each vaccine (and without immunization in the “no vaccine” arm) to be estimated separately and combined based on vaccination coverage. Two comparisons were conducted: aQIV vs. SD-QIV (strategy 1 vs. strategy 2) and aQIV vs. HD-QIV (strategy 1 vs. strategy 3). Considering all clinical events captured in the model were conditional on an influenza infection, the impact of a vaccination strategy on the population (number of events and influenza-related treatment and hospitalization costs) was driven by VE against influenza infection.

### 2.2. Study Population and Analyses Settings

This study focused on adults aged ≥65 years in Denmark, Norway, and Sweden. Modeled populations were further divided into two subgroups (65–74 years and ≥75 years) to account for certain age-specific model inputs. The model projected the health (life years (LYs) and quality adjusted life years (QALYs)) and cost outcomes during one influenza season (assumed to last 6 months), as well as LYs, QALYs, and productivity lost due to premature death resulting from IRCs. Country-specific discount rates for health and cost outcomes (3.5% for Denmark [31,32], 4% for Norway [33], and 3% for Sweden [34]) were used following local pharmacoeconomic guidelines. Healthcare payer and societal perspectives were evaluated in the base case analysis and direct medical costs (vaccination acquisition and administration costs), medical attention (visit to a general practitioner (GP)) costs, costs for IRC complication management, and indirect costs (costs for productivity loss, transportation, and non-prescription medications) were considered in the model.

### 2.3. Model Inputs

#### 2.3.1. Influenza Attack Rate

Reliable estimates of influenza attack rates in the whole population (i.e., proportion of the population that become infected with influenza) are difficult to obtain due to limited testing, especially in asymptomatic patients. Somes et al. have conducted a multi-country systematic review and meta-analysis of vaccine randomized controlled trials (RCT) that reported on laboratory-confirmed seasonal influenza in the placebo arm [35]. They estimated the pooled symptomatic attack rate for all influenza to be 7.2% (95% confidence interval [CI]: 4.3–12.0%) among adults aged ≥65 years, based on three RCTs including 2589 participants. To reflect the attack rate in an average influenza season (i.e., average between severe and mild seasons), we have used 7.2% as the influenza attack rate among the unvaccinated population (Table 1). This is in line with other cost effectiveness studies of influenza vaccines (which have used attack rates ranging from 5% to 10%) [36,37,38] and the WHO estimate of the annual global influenza attack rate in all adults (between 5% and 10%) [3]. Therefore, we have tested 5% and 10% in a sensitivity analysis.

#### 2.3.2. Vaccination Strategy and Coverage Rates

The population aged ≥65 years were vaccinated with aQIV, SD-QIV, or HD-QIV. Vaccine coverage rates were assumed to be the same for the three strategies and varied by country. Non-vaccinated patients (i.e., 100%—coverage rate) were included in the “No vaccine” arm of the model. In the base case, vaccine coverage rates for the 2020/21 season were used for three countries (Table 2). These were used to reflect vaccination coverage rates in the post-coronavirus disease 2019 (COVID-19) pandemic. Scenario analyses to evaluate more conservative vaccine coverage rates were conducted.

#### 2.3.3. Vaccine Effectiveness

The overall effectiveness of SD-QIV was calculated as a weighted average of strain-specific VE and influenza strain circulation information (Table 1). The VE of SD-QIV by influenza subtype (strain) for older adults was sourced from Belongia et al. (H1N1: 62%, H2N3: 24%, B: 63%; Observational data) [39]. To account for seasonal variability and capture VE in an average influenza season, we calculated the average strain circulation for each country and for five influenza seasons prior to the COVID-19 pandemic (2014/15–2018/19) and used the derived SD-QIV effectiveness in the model. We then derived the VEs of aQIV and HD-QIV using the relative efficacy (for HD-QIV vs. SD-QIV) and effectiveness (for aQIV vs. HD-QIV and aQIV vs. SD) between vaccines in preventing influenza infection. Evidence on rVEs for quadrivalent vaccines is limited, and it was assumed that the rVEs between different quadrivalent vaccines were equivalent to those between the respective trivalent formulations. The vaccine efficacy for HD-TIV vs. TIV as reported by Diaz Granados et al. (24.2%; RCT data) was used to estimate the VE of HD-QIV [40]. In turn, the relative effectiveness for aTIV vs. HD-QIV reported by Coleman et al. (3.2%; Observational data) was used to estimate the VE of aQIV [41] (Table 1).

#### 2.3.4. Clinical Inputs

Clinical inputs were sourced from published literature. When available, we sourced evidence originating from Denmark, Norway, and Sweden. In the absence of data from these countries, we used evidence from other countries, giving preference to large studies used to inform previous influenza cost-effectiveness models reported in peer-reviewed publications. All clinical inputs were validated based on the authors’ clinical experience to ensure they were applicable to Denmark, Norway, and Sweden (Table 1 and Table S1). In the model, 66.9% of patients infected with influenza will develop influenza-like symptoms, based on a previous influenza model [30,42]. Among patients with symptomatic influenza, we assumed that 30% would seek medical attention (i.e., a GP visit) [21], based on evidence from a Danish health technology assessment for influenza vaccines [21]. In the model, a variety of respiratory or non-respiratory IRCs could develop in symptomatic patients. The IRCs considered in the model were based on published studies reporting the risk of IRCs in patients with influenza and IRCs included in previous influenza models, and include bronchitis, pneumonia, and any unspecified upper respiratory tract infection (URTI), myocarditis, myocardial infarction (MI), renal complications, central nervous system (CNS) complications, stroke, heart failure (HF), and gastrointestinal bleeding (GI) [29,30]. Risk of the IRCs included in the model were based on [30] a United Kingdom population database analysis including 141,293 subjects diagnosed with influenza or influenza-like illness [105], along with other published studies [44,106,107]. These data were used to inform probability of IRCs in several cost-effectiveness models for influenza. [30].

Patients with IRCs were assumed to require either hospitalization or treatment in outpatient settings. The probabilities of hospitalization for each complication were sourced from published literature [45,46,47,48]. It was assumed that patients that were not hospitalized would be treated in outpatient settings. Patients experiencing an IRC had an increased mortality risk specific to that complication and their age group, regardless of their influenza vaccination history (Table S1). Probabilities of dying after onset of each IRC were sourced from published literature [30,49,50,51,108,109,110,111,112,113].

#### 2.3.5. Health-Related Quality of Life Inputs

The model applied general population utilities and utility decrements for influenza symptomatic infection and IRCs to estimate quality adjusted life years (QALYS) for the different vaccination strategies. Age-specific utility values for the general population in three countries were collected from published literature [103,104]. Utility decrements associated with symptomatic influenza infection were derived by multiplying the utility loss for influenza infection by the duration of symptomatic influenza infection (Table 2). Similarly, utility decrements for IRC-related hospitalizations and outpatient treatment were derived using the disutility and duration associated with each IRC (except for MI and stroke where event disutilities were used) [30]. To account for the longer disease course and recovery time of hospitalized influenza patients compared to outpatient cases, disutilities for three days of outpatient care were added to the total utility loss applied to hospitalizations (Table S1). Utility decrements of next-of-kin were not included in the model.

#### 2.3.6. Costs and Resource Use Inputs

A summary of costs used in the base case is provided in Table 1. All costs reported in previous years were inflated to 2022 euros using country-specific consumer price indices [114,115,116]. Real vaccine prices are difficult to accurately estimate as vaccines are usually purchased directly from manufacturers through tenders and other confidential negotiations. In our analysis, we sought to use vaccine prices as close as possible to the net cost for the health authorities. As such, SD-QIV prices were sourced from a third-party price database (IQVIA) which estimated unit costs from sales data for each country [82]. As we could not identify net HD-QIV prices in the relevant countries, we derived the price for HD-QIV based on the maximum price per dose referenced in publicly available records of contracts and tenders for the 2021/22 and 2022/23 influenza seasons in Italy and Spain [83,84]. Both countries have a similar vaccine procurement process to Denmark, Norway, and Sweden. The aQIV prices are presented as a ratio versus SD-QIV prices and were based on the net price anticipated by the manufacturer (Table 2). Vaccines were assumed to be administered during a GP visit and the cost of vaccine administration assumed equal to the cost of a GP visit (except for Sweden, in which we assumed the cost of a nurse appointment). Inpatient and outpatient costs for IRCs were sourced from official diagnosis-related group listings (Table S2). Official statistics from Denmark, Norway, and Sweden show that 15 to 20% of individuals aged over 65 years are employed [90,91,92]. Therefore, the model considered indirect costs due to productivity loss. We estimated absenteeism based on the working days lost due to influenza illness and IRCs as well as productivity loss due to premature death due to influenza IRCs using the human capital approach [117]. These costs were estimated for the proportion of working individuals in the 65–74 years age group (Table 1) and no productivity loss costs were assumed for the population aged ≥75 years. The working days lost for symptomatic influenza cases and each IRC leading to hospitalization were sourced from published literature (Table 1 and Table S1). For each IRC treated in outpatient settings, we assumed half of the days lost when the complication led to hospitalization.

### 2.4. Cost-Effectiveness Analysis

The base case analyses included two comparisons: aQIV vs. SD-QIV and aQIV vs. HD-QIV. The following outcomes were evaluated: numbers of clinical events (symptomatic influenza cases, GP visits, IRCs, hospitalizations, and influenza-related deaths), costs (total cost, cost of vaccine, cost of vaccine administration, cost of GP visits, cost of IRCs, and societal costs), life years (LY) and quality adjusted life years (QALY), ICERs, and net monetary benefit (NMB). In addition, the model estimated the maximum price ratio of aQIV (vs. SD-QIV) at which aQIV remained a cost-effective strategy.

There are no official willingness to pay thresholds in Denmark, Norway, and Sweden. We identified economic evaluations in these countries that use different thresholds ranging from EUR 22,000 to EUR 55,000 per QALY [118,119,120]. To allow easy interpretation of the model results and enable a comparison between the three countries, we adopted a commonly referenced threshold in Europe (~EUR 30,000/QALY) [28,121].

### 2.5. Scenario and Sensitivity Analyses

Scenario analyses were conducted to evaluate the robustness of the model results by changing input parameters and/or assumptions using alternative data sources and hypotheses (Table 3). Univariate deterministic sensitivity analysis (DSA) was conducted using available 95% CIs for model parameters, or a ±20% variation around the base case value. The joint uncertainty of the model was assessed in probabilistic sensitivity analysis (PSA) using second-order Monte Carlo simulations (1000 iterations).

## 3. Results

### 3.1. Base Case Results

The base case results for the comparison between aQIV and SD-QIV are presented in Table 4. Use of aQIV instead of SD-QIV resulted in incremental costs of around EUR 4.7 M for Denmark, EUR 3.6 M for Norway, and EUR 6.3 M for Sweden. For all countries, the increased costs associated with vaccination were partially offset by cost savings in GP visits and management of IRCs. The model predicts that the increased cost associated with aQIV can result in considerable health benefits for the population. In Denmark, aQIV could prevent 6238 symptomatic influenza cases, 1871 GP visits, 307 hospitalizations, and 54 deaths in an average influenza season. For Norway, the model predicts that 3810 symptomatic influenza cases, 1143 GP visits, 187 hospitalizations, and 32 deaths would be avoided per season. For Sweden, our model projects a reduction of 8724 symptomatic influenza cases, 2617 GP visits, 431 hospitalizations, and 75 deaths. Based on our analyses, the aQIV was a cost-effective strategy compared to SD-QIV in the three countries from healthcare payer and societal perspectives. For a threshold of EUR 30,000/QALY and the healthcare payer perspective, aQIV remained a cost-effective vaccination strategy if the price ratio vs. SD-QIV did not exceed 293% in Denmark, 306% in Norway, and 286% in Sweden.

When compared to HD-QIV, our analysis suggests that aQIV is a cost-saving vaccination strategy, resulting in savings of around EUR 7.1 M in Denmark, EUR 4.7 M in Norway, and EUR 8.5 M in Sweden (Table 5). The cost difference was mainly due to the lower vaccine acquisition costs assumed for aQIV in the analysis. Since aQIV and HD-QIV were assumed to have comparable VE, the health benefits in favor of aQIV were marginal for this comparison.

### 3.2. Scenario Analysis

Results from the scenario analyses conducted for the comparison of aQIV vs. SD-QIV (from a healthcare payer perspective) are presented in Table 6. Scenario analyses for the societal perspective and for the comparison against HD-QIV are provided in Supplementary Materials (Tables S3–S5).

Variations in the vaccine coverage rate (scenario 1) had a negligible impact on incremental outcomes of the model (and consequently ICERs) since the variation in coverage rate was equally applied to both strategies. The analysis using influenza strain distribution from individual seasons (scenarios 2.1–2.5), yielded ICERs ranging between EUR 5650 and EUR 17,354 with aQIV remaining a cost-effective strategy in all scenarios across all countries. When using only observational data to estimate the rVE of aQIV (scenario 3), the ICERs increased in all countries, but aQIV remained a cost-effective strategy. When varying the strain-specific VE of SD-QIV (scenarios 4.1 and 4.2), results ranged from aQIV being a dominant strategy (in all countries) to a maximum ICER of EUR 21,615/QALY. When varying rVEs for HD-QIV vs. SD-QIV and aQIV vs. HD-QIV (scenarios 5.1 and 5.2), we observed a significant impact on the ICERs for aQIV vs. SD-QIV, ranging from dominant to ICERs above the assumed cost-effectiveness threshold in all countries. The impact of the management costs of IRCs (scenarios 6.1 and 6.2) was moderate and ICERs varied between ±35% and ±43% from base case results when all complication-related costs were varied by ±30%. When excluding HF as an IRC (scenario 7), ICERs showed a small increase with aQIV remaining a cost-effective strategy in all countries.

### 3.3. DSA (Healthcare Payer Perspective)

**Figure 2:**
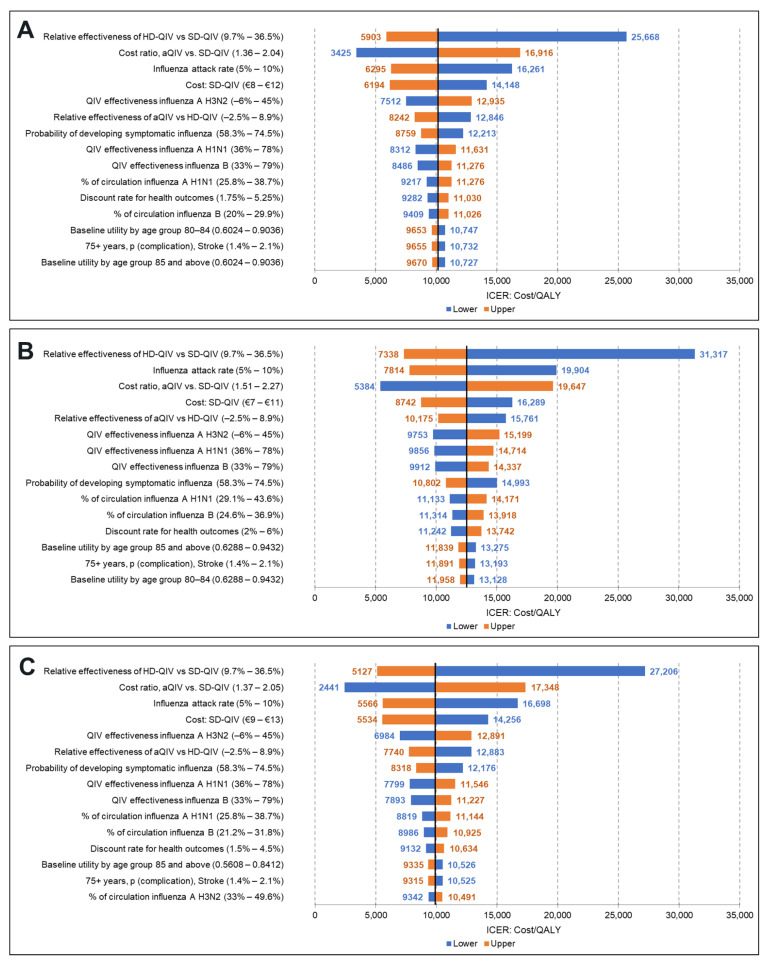
DSA results (aQIV vs. SD-QIV): (**A**) Denmark, (**B**) Norway, (**C**) Sweden.

Results of the DSA are presented in Figure 2, reporting the 15 parameters with the highest impact on the ICER for the aQIV vs. SD-QIV comparison. Tornado diagrams for the societal perspective and comparison of aQIV vs. HD-QIV are provided in the Supplementary Materials (Figures S3–S5).

Variations on the rVE of HD-QIV vs. SD-QIV (9.7–36.5%) and aQIV vs. HD-QIV (−2.5–8.9%), strain-specific VE of SD-QIV against H3N2 (−6–45%), influenza attack rate (5–10%), and acquisition costs of both vaccines were the major drivers of the ICER for the three countries. The remaining parameters in the model had a moderate to minor impact on the ICERs. The aQIV remained a cost-effective strategy in all but one of the scenarios tested in the DSA (lower bound of rVE between aQIV and HD-QIV for Norway).

### 3.4. PSA (Healthcare Payer Perspective)

**Figure 3:**
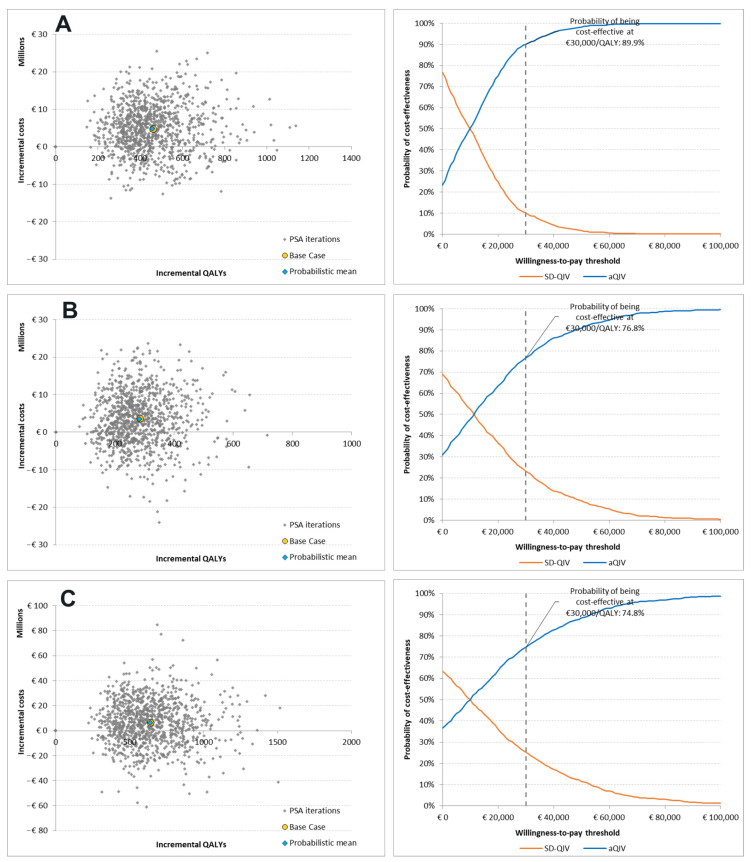
PSA results (aQIV vs. SD-QIV): (**A**) Denmark, (**B**) Norway, and (**C**) Sweden.

PSA results (scatter plots and cost-effectiveness acceptability curves) for aQIV vs. SD-QIV are presented in Figure 3. PSA results for the societal perspective and comparison between aQIV and HD-QIV are provided in Supplementary Materials (Figures S4–S6).

From a healthcare payer perspective, the cost-effectiveness acceptability curves (CEACs) showed that aQIV was more likely to be cost-effective than SD-QIV in Denmark (90%), Norway (77%), and Sweden (75%), at a threshold of EUR 30,000/QALY. Our results showed that aQIV was a dominant strategy in 23%, 32%, and 36% of the simulations for Denmark, Norway, and Sweden, respectively.

## 4. Discussion

Due to immunosenescence, SD-QIV provides limited protection for older adults. Lower vaccine effectiveness and higher prevalence of comorbidities, result in higher influenza-related morbidity and mortality among this age group (≥65 years). Enhanced vaccines such as the aQIV and HD-QIV elicit increased immune response among this population and have demonstrated improved VE compared to the SD-QIV [16,40,41]. Although enhanced vaccines are recommended for specific high-risk groups in Norway and Sweden, evidence is lacking for the cost-effectiveness of extending influenza vaccination with aQIV to the entirety of the population aged ≥65 years.

In this study, we developed a static decision-tree model to estimate the influenza-related disease and economic burden based on different vaccination strategies in Denmark, Norway, and Sweden for adults ≥65 years. To our knowledge, this is the first cost-effectiveness study of aQIV in these countries. The model results indicate that aQIV can prevent a higher number of symptomatic influenza cases, influenza-related complications leading to hospitalizations, and deaths compared to SD-QIV. Vaccination with aQIV led to cost increases and QALY gains and aQIV was a cost-effective vaccination strategy for adults aged ≥65 years compared to SD-QIV. In comparison to HD-QIV, aQIV resulted in cost savings and comparable health benefits in the three countries.

Our results align with previous modeling studies for influenza vaccination strategies with aQIV in other European countries. Fochesato et al. showed that replacing SD-QIV with aQIV in the Spanish adult population aged ≥65 years was a cost-effective strategy based on a compartmental disease transmission model and decision-tree model [25]. Kohli et al. used a similar modeling approach and confirmed that aQIV was a cost-saving alternative to HD-QIV in adults aged ≥65 years in Germany and the UK [26,27]. Ruiz-Aragon et al. used a static, decision-tree model to compare the strategy of vaccinating the Spanish population aged ≥65 years with HD-QIV or aQIV and showed that aQIV was a cost-saving alternative to HD-QIV [24].

The disease burden projected in our analysis is conservative when compared with the available evidence in Denmark, Norway, and Sweden. The Public Health Agency of Sweden reported that there were between 206 and 949 deaths from influenza each year between seasons 2015/16 and 2018/19 compared to 636 deaths projected in our study for Sweden (for the SD-QIV strategy) [77,79,80,81]. The Danish Health Authority reported the number of patients aged ≥65 years hospitalized with influenza to vary between 968 and 4771, and the number of influenza-related deaths to vary from 332 to 1391 between seasons 2015/16 and 2018/19 [122]. In the SD-QIV strategy, our analysis projected 1900 influenza-associated hospitalizations and 320 deaths in Denmark. In Norway, the number of hospitalized influenza patients was estimated to be between 579 and 4973 per season [123,124], and influenza-related excess mortality to be 910 per season [125]. Our results showed 1600 hospitalizations and 280 deaths due to influenza in Norway. We identified several reasons to explain the lower estimates of the burden of influenza projected by our model. First, we assumed vaccination coverage from the 2020/21 influenza season, which was higher than previous years. In addition, the list of IRCs is not exhaustive and there may be additional complications attributable to influenza that were not captured in our model. Furthermore, the incidence and mortality risk of cardiovascular complications were considered low based on the authors’ clinical experience. Finally, our model did not account for IRCs that may develop in asymptomatic or unreported influenza infections.

Our base case model findings were robust based on several sensitivity and scenario analyses. However, model results were sensitive to the inputs and assumptions used to inform effectiveness of the vaccines included in the analyses. Strain-specific effectiveness for SD-QIV and rVEs for the enhanced vaccines were key drivers of the model results. This is a result of the wide confidence intervals in the estimates sourced from the literature. For instance, the rVE of aQIV vs. HD-QIV ranges from negative to positive values, indicating non-statistically significant estimates that suggest comparability. Nevertheless, DSA and scenario analyses on these model parameters showed that aQIV remained a cost-effective strategy in most alternative scenarios evaluated. The influenza attack rate was another driver of the model outcomes, showing an increase in the value of vaccination with aQIV during severe influenza seasons. PSA showed that aQIV has a high likelihood of being a cost-effective or dominant vaccination strategy in comparison with SD-QIV and HD-QIV.

The findings of this study must be interpreted considering several limitations. There is no evidence from randomized controlled trials (RCT) or observational studies directly comparing the effectiveness of standard-dose quadrivalent vaccines with the respective enhanced formulations (aQIV and HD-QIV). Hence, we have used evidence from the trivalent formulations. It should be noted that effectiveness evidence from studies from the trivalent formulations are relevant for QIV vaccines as they have overlapping compositions and the same manufacturing process [126]. Furthermore, RCT evidence was only available to inform the efficacy of HD compared to SD vaccines. Hence, we used effectiveness data from a meta-analysis of observational studies to inform the rVE of the adjuvanted versus high-dose vaccine. The analyses did not include vaccine-related adverse events. This is aligned other economic evaluations of enhanced influenza vaccines and is unlikely to affect our findings [24,25,26,27]. While a higher reactogenicity is expected for aQIV vs. SD-QIV, we do not expect a significant clinical impact, as this results mostly in localized reactions which usually do not require medical care [126,127].

Country-specific evidence was limited on the incidence of IRCs and IRC-related hospitalization and mortality with the granularity required for our model. We have used inputs derived from other countries and validated their applicability to Denmark, Norway, and Sweden based on the authors’ clinical experience. We decided to include HF as an IRC in the model base case, as this is the “end disease” for all cardiovascular conditions. Some evidence suggests that influenza vaccination has associated benefits in terms of CV disease outcomes. A recent meta-analysis reported lower risks of all-cause and CV-related mortality for HF patients vaccinated against influenza [128]. In addition, a randomized feasibility trial showed that HD-QIV was associated with lower hospitalization and all-cause mortality rates compared with SD-QIV [16]. However, no evidence was identified to inform the risk of developing HF after influenza infection. Hence in the base case, we assumed that the risk of HF among symptomatic influenza patients was the same as for myocarditis. Acknowledging the uncertainty around the risk of HF due to influenza infection, we conducted a scenario analysis that excluded HF from the model. We noted a small increase in the ICER, but our conclusions were not affected since aQIV remained a cost-effective (vs. SD-QIV) or cost-saving (vs. HD-QIV) strategy.

Another limitation is related to the net prices for the different vaccines in Denmark, Norway, and Sweden. Due to confidential commercial agreements and tenders negotiated directly with manufacturers, we could not accurately estimate net prices. Therefore, we used publicly available evidence from other European countries to inform our price assumptions. DSAs showed that vaccine prices were among the main drivers of model outcomes. Nevertheless, our findings remained consistent for variations of ±20% in the prices of all vaccines.

Future vaccine coverage rates are an area of uncertainty. Vaccination rates in the three countries have been affected by the COVID-19 pandemic. Compared with previous years, higher coverage rates in the 2020/21 influenza season were reported likely due to increased public awareness for the consequences of respiratory infections. We used coverage rates from the 2020/21 season under the assumption that influenza vaccination rates will remain high for the following years (especially in high-risk patients such as the elderly). Nevertheless, to address the uncertainty around this parameter, we conducted a scenario analysis with lower vaccine coverage rates and aQIV remained a cost-effective (vs. SD-QIV) or cost-saving (vs. HD-QIV) strategy.

Finally, our study used a static model that did not capture the dynamics of disease transmission, such as impact of seasonal variations, interactions between individuals, and herd immunity (i.e., indirect protection of unvaccinated individuals when a significant portion of a population is vaccinated). Incorporating herd immunity would likely show additional benefits in favor of the most effective vaccines (i.e., aQIV and HD-QIV) [129].

## 5. Conclusions

Our analyses indicated that, in an average influenza season, aQIV may be a cost-effective strategy compared to SD-QIV and may be cost-saving when compared to HD-QIV for preventing seasonal influenza among adults aged ≥65 years in Denmark, Norway, and Sweden. The introduction of aQIV may prevent a significant number of influenza cases and IRCs, leading to a lower disease burden for patients and reducing the economic burden for healthcare payers and society.

## Figures and Tables

**Table 1:** Model inputs applied across all countries.

Parameter	Value	Reference
Influenza attack rate in unvaccinated population	7.20%	[35]
Vaccine effectiveness		
SD-QIV VE against influenza A H1N1	62.00%	[39]
SD-QIV VE against influenza A H3N2	24.00%	
SD-QIV VE against influenza B	63.00%	
rVE HD-QIV vs. SD-QIV	24.20%	[40]
rVE aQIV vs. HD-QIV	3.20%	[41]
Clinical events		
Probability of developing symptomatic influenza	66.90%	[42]
Probability of medical attention (GP visit)	30.00%	[21]
Probability of developing IRCs	IRC specific (see Table S1)	[30,43,44]
Probability of hospitalization due to IRCs		[45,46,47,48]
Probability of death due to IRCs		[30,49,50,51]
Societal perspective inputs		
Number of days lost due to symptomatic influenza	3.20	[52]
Number of days lost due to hospitalization	IRC specific (see Table S1)	[53,54,55,56,57,58,59,60]
Utility decrements		
Symptomatic influenza	−0.0079	[30]
Hospitalization	IRC specific (see Table S1)	[30]
Outpatient		[30]

aQIV: Adjuvanted quadrivalent influenza vaccine; GP: General practitioner; HD-QIV: High-dose quadrivalent influenza vaccine; IRCs: Influenza-related complications; QIV: Quadrivalent influenza vaccine; rVE: Relative vaccine effectiveness; VE: Vaccine effectiveness.

**Table 2:** Country-specific model inputs.

Parameter	Denmark	Norway	Sweden	References
Population and vaccination strategy				
Population size (≥65 years)	1,198,676	989,474	2,118,766	[61,62,63]
Proportion of males (%)	46.10%	46.90%	46.70%	
Vaccine coverage rate	75.00%	59.70%	60.00%	[64,65,66]
Influenza strain distribution ^a^				
Influenza A (H1N1)	32.20%	36.30%	32.20%	[67,68,69,70,71,72,73,74,75,76,77,78,79,80,81]
Influenza A (H3N2)	42.80%	32.90%	41.30%	
Influenza B	25.00%	30.70%	26.50%	
Vaccine costs (€)				
SD-QIV	10.20	9.10	11.00	[82]
aQIV (ratio vs. SD-QIV)	170%	189%	171%	Assumption
HD-QIV	25.00	25.00	25.00	[83,84]
Vaccine admin. costs (€)	30.47	54.81	68.10	
Direct medical costs (€)				
GP visit	30.47	54.81	167.11	[85,86]
Hospitalization	IRC specific (see Table S2)			[86,87,88]
Outpatient				[86,88,89]
Societal perspective inputs				
Proportion of the population employed (65–74 years)	16.6%	19.7%	19.2%	[90,91,92]
Labor costs per day (EUR)	309.13	250.68	294.40	[93,94,95]
Non-prescription medication (EUR)	4.30	2.61	1.03	[96,97,98]
Transport (vaccination) (EUR)	5.03	2.65	4.84	[99,100,101,102]
Transport (outpatient) (EUR)	5.03	2.65	4.84	
Transport (hospitalization) (EUR)	14.84	7.66	4.84	
Baseline utilities				
65–69 years	0.82	0.80	0.84	[103,104]
70–74 years	0.82	0.80	0.84	
75+ years	0.75	0.79	0.70	

1 EUR = 10.18 NOK/7.44 DKK/10.68 SEK; ^a^ Average of seasons 2014/15 to 2018/19; aQIV: Adjuvanted quadrivalent influenza vaccine; GP: General practitioner; HD-QIV: High-dose quadrivalent influenza vaccine; IRC: Influenza-related complication; SD-QIV: Standard-dose quadrivalent influenza vaccine.

**Table 3:** Scenario analyses.

Scenario	Description	References
Scenario 1	Assumed lower vaccine coverage rates (47.6% for Denmark, 29.5% for Norway, and 50% for Sweden, average coverage from seasons 2014/15 to 2018/19)	[64,65,77,78,79,80,81]
Scenario 2 (2.1 to 2.5)	Conducted separate analyses for seasons 2014/15 to 2018/19 (5 seasons before COVID-19 pandemic) with influenza strain circulation for each season	[67,68,69,70,71,72,73,74,75,76,77,78,79,80,81]
Scenario 3	aQIV VE estimated using only effectiveness evidence from observational studies (using rVE for aTIV vs. SD-TIV from Coleman et al. [13.9%]) [41]	[41]
Scenario 4 (4.1 to 4.2)	Used lower (36% for H1N1, −6% for H3N2, and 33% for subtype B) and upper (78% for H1N1, 45% for H3N2, and 79% for subtype B) bounds of the 95% CI for SD-QIV VE	[39]
Scenario 5 (5.1 to 5.2)	Used lower and upper bounds of the 95% CI for rVE of HD-QIV vs. SD-QIV (9.7–36.5%) and aQIV vs. HD-QIV (−2.5–8.9%)	[40,41]
Scenario 6 (6.1 to 6.2)	Varied the management costs of all IRCs (outpatient and hospitalizations) by ±30%	NA
Scenario 7	Removed HF from the model	NA

aQIV: Adjuvanted quadrivalent influenza vaccine; HD-QIV: High-dose quadrivalent influenza vaccine; HF: Heath failure; IRC: Influenza-related complications; SD-QIV: Standard-dose quadrivalent influenza vaccine; rVE: Relative vaccine effectiveness; VE: Vaccine effectiveness.

**Table 4:** Model results (aQIV vs. SD-QIV).

	Denmark			Norway			Sweden		
	aQIV	SD-QIV	Incremental	aQIV	SD-QIV	Incremental	aQIV	SD-QIV	Incremental
Population size (≥65 years)	1,198,676			989,474			2,118,766		
**Health outcomes**									
Number of symptomatic influenza cases	31,590	37,828	−6238	29,685	33,495	−3810	64,815	73,539	−8724
Number of GP visits	9477	11,348	−1871	8906	10,048	−1143	19,445	22,062	−2617
Number of IRCs	5009	5998	−989	4700	5303	−603	10,287	11,672	−1385
Number of hospitalizations	1554	1861	−307	1454	1640	−187	3199	3629	−431
Number of deaths	271	325	−54	252	285	−32	560	636	−75
Total LYs	12,115,917	12,115,416	501	10,000,047	9,999,745	301	22,394,455	22,393,722	732
Total QALYs	9,325,198	9,324,737	461	7,902,627	7,902,342	285	16,430,639	16,429,998	641
**Costs outcomes (EUR)**									
Total costs (healthcare payer)	51,515,927	46,825,793	4,690,134	52,035,223	48,469,152	3,566,071	136,996,364	130,650,741	6,345,624
Cost of vaccines	15,552,821	9,169,871	6,382,950	10,160,315	5,375,515	4,784,799	23,899,680	13,983,856	9,915,825
Cost of vaccine administration	27,390,008	27,390,008	0	32,378,525	32,378,525	0	86,570,351	86,570,351	0
Cost of GP visits	288,737	345,750	−57,013	488,136	550,781	−62,645	3,249,334	3,686,664	−437,331
Cost of IRCs (outpatient)	1,157,037	1,385,502	−228,465	712,610	804,063	−91,454	2,572,916	2,919,207	−346,291
Cost of IRCs (hospitalization)	7,127,324	8,534,662	−1,407,338	8,295,638	9,360,267	−1,064,629	20,704,084	23,490,664	−2,786,580
Total costs (societal)	67,020,197	64,496,697	2,523,500	63,835,993	61,583,322	2,252,671	167,147,145	164,032,551	3,114,594
Societal costs	15,504,270	17,670,904	−2,166,634	11,800,770	13,114,170	−1,313,400	30,150,780	33,381,810	−3,231,029
**Cost effectiveness outcomes (Healthcare payer perspective)**									
ICER (EUR /QALY)	10,170			12,515			9894		
NMB (EUR)	9,144,058			4,981,874			12,893,567		
**Cost effectiveness outcomes (Societal perspective)**									
ICER EUR /QALY)	5472			7906			4856		
NMB (EUR)	11,310,692			6,295,273			16,124,596		

1 EUR = 10.18 NOK/7.44 DKK/10.68 SEK; aQIV: Adjuvanted quadrivalent influenza vaccine; GP: General practitioner; ICER: Incremental cost-effectiveness ratio; IRCs: Influenza-related complications; LY: Life year; SD-QIV: Standard-dose quadrivalent influenza vaccine; NMB: Net monetary benefit; QALY: Quality adjusted life year.

**Table 5:** Model results (aQIV vs. HD-QIV).

	Denmark			Norway			Sweden		
	aQIV	HD-QIV	Incremental	aQIV	HD-QIV	Incremental	aQIV	HD-QIV	Incremental
Population size (≥65 years)	1,198,676			989,474			2,118,766		
**Health outcomes**									
Number of symptomatic influenza cases	31,590	32,157	−567	29,685	30,032	−346	64,815	65,608	−793
Number of GP visits	9477	9647	−170	8906	9009	−104	19,445	19,683	−238
Number of IRCs	5009	5099	−90	4700	4755	−55	10,287	10,413	−126
Number of hospitalizations	1554	1582	−28	1454	1471	−17	3199	3238	−39
Number of deaths	271	276	−5	252	255	−3	560	567	−7
Total LYs	12,115,917	12,115,871	46	10,000,047	10,000,019	27	22,394,455	22,394,388	67
Total QALYs	9,325,198	9,325,156	42	7,902,627	7,902,601	26	16,430,639	16,430,581	58
**Costs outcomes (EUR)**									
Total costs (healthcare payer)	51,515,927	58,592,192	−7,076,265	52,035,223	56,753,615	−4,718,391	136,996,364	145,202,777	−8,206,412
Cost of vaccines	15,552,821	22,475,175	−6,922,354	10,160,315	14,767,899	−4,607,585	23,899,680	31,781,490	−7,881,810
Cost of vaccine administration	27,390,008	27,390,008	0	32,378,525	32,378,525	0	86,570,351	86,570,351	0
Cost of GP visits	288,737	293,920	−5184	488,136	493,832	−5696	3,249,334	3,289,096	−39,762
Cost of IRCs (outpatient)	1,157,037	1,177,809	−20,772	712,610	720,925	−8315	2,572,916	2,604,400	−31,485
Cost of IRCs (hospitalization)	7,127,324	7,255,279	−127,955	8,295,638	8,392,434	−96,796	20,704,084	20,957,439	−253,356
Total costs (societal)	67,020,197	74,293,452	−7,273,255	63,835,993	68,673,799	−4,837,806	167,147,145	175,647,322	−8,500,177
Societal costs	15,504,270	15,701,260	−196,990	11,800,770	11,920,184	−119,414	30,150,780	30,444,546	−293,765
**Cost effectiveness outcomes (Healthcare payer perspective)**									
ICER (EUR /QALY)	Dominant			Dominant			Dominant		
NMB (EUR)	8,334,069			5,495,571			9,955,639		
**Cost effectiveness outcomes (Societal perspective)**									
ICER EUR /QALY)	Dominant			Dominant			Dominant		
NMB (EUR)	8,531,060			5,614,985			10,249,405		

1 EUR = 10.18 NOK/7.44 DKK/10.68 SEK; aQIV: Adjuvanted quadrivalent influenza vaccine; GP: General practitioner; ICER: Incremental cost-effectiveness ratio; IRCs: Influenza-related complications; HD-QIV: High-dose quadrivalent influenza vaccine; LY: Life year; NMB: Net monetary benefit; QALY: Quality adjusted life year.

**Table 6:** Scenario analysis results (aQIV vs. SD-QIV).

Scenario	ICER (EUR/QALY)		
	Denmark	Norway	Sweden
*Base case*	10,170	12,515	9894
Scenario 1 (Vaccine coverage rates)	10,170	12,515	9894
Scenario 2.1 (Strain distribution 2014/15)	9364	11,522	9162
Scenario 2.2 (Strain distribution 2015/16)	15,263	17,354	15,507
Scenario 2.3 (Strain distribution 2016/17)	6275	7966	5650
Scenario 2.4 (Strain distribution 2017/18)	12,952	14,563	12,291
Scenario 2.5 (Strain distribution 2018/19)	10,412	14,422	10,333
Scenario 3 (aQIV rVE vs. SD-QIV at 13.9%)	22,881	27,936	24,093
Scenario 4.1 (lower bound SD-QIV VE)	5367	6429	4490
Scenario 4.2 (upper bound SD-QIV VE)	17,168	21,615	17,793
Scenario 5.1 (lower bound rVEs)	45,919	55,886	49,828
Scenario 5.2 (upper bound rVEs)	5085	6345	4213
Scenario 6.1 (30% decrease in complication costs)	11,234	13,732	11,360
Scenario 6.2 (30% increase in complication costs)	9106	11,298	8429
Scenario 7 (exclude HF)	12,318	14,998	11,977

1 EUR = 10.18 NOK/7.44 DKK/10.68 SEK; aQIV: Adjuvanted quadrivalent influenza vaccine; HF: Heart failure; ICER: Incremental cost-effectiveness ratio; QALY: Quality adjusted life year; SD-QIV: Standard-dose quadrivalent influenza vaccine; rVE: Relative vaccine effectiveness; VE: Vaccine effectiveness.

## Data Availability

The data presented in this study are available within the article or in the Supplementary Materials.

## Supplementary Materials

The following supporting information can be downloaded at https://www.mdpi.com/article/10.3390/vaccines11040753/s1, Table S1: Clinical, societal, and utility loss inputs for IRCs, Table S2: Hospitalization and outpatient treatment costs for IRCs, Table S3: Scenario analysis results (aQIV vs. SD-QIV)—Societal perspective; Table S4: Scenario analysis results (aQIV vs. HD-QIV)—Healthcare payer perspective; Table S5: Scenario analysis results (aQIV vs. HD-QIV)—Societal perspective; Figure S1: DSA results (aQIV vs. SD-QIV)—Societal perspective: (A) Denmark, (B) Norway, (C) Sweden; Figure S2: PSA results (aQIV vs. SD-QIV)—Societal per-spective: (A) Denmark, (B) Norway, and (C) Sweden; Figure S3: DSA results (aQIV vs. HD-QIV)—Healthcare payer perspective: (A) Denmark, (B) Norway, (C) Sweden; Figure S4: PSA results (aQIV vs. HD-QIV)—Healthcare payer perspective: (A) Denmark, (B) Norway, and (C) Sweden; Figure S5: DSA results (aQIV vs. HD-QIV)—Societal perspective: (A) Denmark, (B) Norway, (C) Sweden; Figure S6: PSA results (aQIV vs. HD-QIV)—Societal perspective: (A) Den-mark, (B) Norway, and (C) Sweden. References [30,43,44,45,46,47,48,49,50,51,53,54,55,56,57,58,59,60,86,87,88,89] are cited in the supplementary materials.
