# Supplementary material for: Cost-Effectiveness of Vaccination of Older Adults with an MF59®-Adjuvanted Quadrivalent Influenza Vaccine Compared to Standard-Dose and High-Dose Vaccines in Denmark, Norway, and Sweden
Source: Vaccines (Basel). 2023 Mar 29;11(4):753. doi: 10.3390/vaccines11040753 (PMC10145635; doi:10.3390/vaccines11040753)
Supplement: Supplementary file 1 [file vaccines-11-00753-s001.zip › vaccines-2280680-supplementary.pdf]

Supplementary materials

List of tables

Table S1. Clinical, societal, and utility loss inputs for IRCs
Table S2. Hospitalization and outpatient treatment costs for IRCs
Table S3. Scenario analysis results (aQIV vs. SD-QIV) – Societal perspective
Table S4. Scenario analysis results (aQIV vs. HD-QIV) – Healthcare payer perspective
Table S5. Scenario analysis results (aQIV vs. HD-QIV) - Societal perspective

List of figures

Figure S1. DSA results (aQIV vs. SD-QIV) – Societal perspective: (A) Denmark, (B) Norway, (C) Sweden
Figure S2. PSA results (aQIV vs. SD-QIV) – Societal perspective: (A) Denmark, (B) Norway, and (C) Sweden
Figure S3. DSA results (aQIV vs. HD-QIV) – Healthcare payer perspective: (A) Denmark, (B) Norway, (C) Sweden
Figure S4. PSA results (aQIV vs. HD-QIV) – Healthcare payer perspective: (A) Denmark, (B) Norway, and (C) Sweden
Figure S5. DSA results (aQIV vs. HD-QIV) – Societal perspective: (A) Denmark, (B) Norway, (C) Sweden
Figure S6. PSA results (aQIV vs. HD-QIV) – Societal perspective: (A) Denmark, (B) Norway, and (C) Sweden

Table S1. Clinical, societal, and utility loss inputs for IRCs.

Parameter	Value[a]	Reference
**Clinical events**		
Probability of developing IRCs		
Bronchitis	3.85%	[1]
Pneumonia	1.46%	
URTI	6.05%	
Myocarditis	0.89%	
Renal complications	0.18%	
CNS complications	0.35%	
GI bleeding	0.81%	
MI	0.13%	
Stroke		
Age 65–74 years	0.75%	[2,3]
Age 75+ years	1.79%	
HF	0.89%	Assumed same as myocarditis
Probability of hospitalization due to IRCs		
Bronchitis	1.00%	[4]
Pneumonia		
Age 65–74 years	75.40%	[5]
Age 75+ years	83.70%	
URTI	1.00%	Assumed same as bronchitis
Myocarditis		
Age 65–74 years	75.40%	Assumed same as pneumonia
Age 75+ years	83.70	
Renal complications	100.00%	Assumption
CNS complications	100.00%	Assumption
GI bleeding	75.90%	[6]
MI	100.00%	Assumption
Stroke	86.00%	[7]
HF		
Age 65–74 years	75.40%	Assumed same as myocarditis
Age 75+ years	83.70%	
Probability of death due to IRCs		
Bronchitis	0.03%	[1]
Pneumonia	6.90%	
URTI	0.03%	
Myocarditis	20.80%	[8]
Renal complications		
Age 65–74 years	14.15%	[1]
Age 75+ years	28.40%	
CNS complications	2.90%	
GI bleeding	3.00%	
MI		
Age 65–74 years	11.90%	[1]
Age 75+ years	22.33%	
Stroke	28.20%	[9]
HF		
Age 65–74 years	11.80%	[10]
Age 75+ years	16.70%	
**Societal perspective inputs**		

Number of days lost due to hospitalization		
Bronchitis	3.20	[11]
Pneumonia	10.00	[12]
URTI	5.40	[13]
Myocarditis	36.00	[14]
Renal complications	10.80	[15]
CNS complications	7.60	[16]
GI bleeding	4.00	[17]
MI	44.00	[18]
Stroke	34.00	[14]
HF	36.00	Assumed same as myocarditis
**Utility decrements**		
Hospitalization		
Bronchitis	–0.0090	[1]
Pneumonia	–0.0116	
URTI	–0.0073	
Myocarditis	–0.0085	
Renal complications	–0.0120	
CNS complications	–0.0088	
GI bleeding	–0.0054	
MI	–0.1980	
Stroke	–0.2870	
HF	–0.0085	Assumed same as myocarditis
Outpatient		
Bronchitis	–0.0046	[1]
Pneumonia	–0.0063	
URTI	–0.0021	
Myocarditis	–0.0025	
Renal complications	–0.0050	
CNS complications	–0.0046	
GI bleeding	–0.0014	
MI	–0.1980	
Stroke	–0.2870	
HF	–0.0025	

CNS: Central nervous system; HF: Hearth failure; IRCs: Influenza-related complications; MI: Myocardial infarction; URTI: Upper respiratory tract infection.

Table S2. Hospitalization and outpatient treatment costs for IRCs.

Parameter	Denmark	Norway	Sweden	References
**Direct medical costs (€)**				
Hospitalization				
Bronchitis	3156.92	3707.20	4588.14	[19-21]
Pneumonia	5386.09	5104.72	5893.72	
URTI	3398.06	3439.89	3793.03	
Myocarditis	2731.76	5104.72	5814.89	
Renal complications	5630.19	6959.50	7360.09	
CNS complications	2656.75	2976.86	3734.70	
GI bleeding	3903.47	4832.72	5765.58	
MI	2668.18	7700.48	5814.89	
Stroke	4940.09	7700.48	9000.28	
HF	6429.13	6664.06	6321.69	

Parameter	Denmark	Norway	Sweden	References
Outpatient				
Bronchitis	297.78	253.24	404.46	[20-22]
Pneumonia	360.18	342.33	527.92	
URTI	286.24	271.38	325.88	
Myocarditis	980.50	215.71	364.94	
Renal complications	499.66	159.43	527.92	
CNS complications	223.55	361.09	462.73	
GI bleeding	558.06	262.67	404.09	
MI	980.50	178.19	423.58	
Stroke	980.50	182.90	430.13	
HF	980.50	178.21	436.69	

1 Euro = 10.18 NOK/ 7.44 DKK/ 10.68 SEK; CNS: Central nervous system; HF: Hearth failure; IRCs: Influenza-related complications; MI: Myocardial infarction; URTI: Upper respiratory tract infection.

Table S3. Scenario analysis results (aQIV vs. SD-QIV) – Societal perspective.

Scenario	ICER (€/QALY)		
	Denmark	Norway	Sweden
Base case	5472	7906	4856
Scenario 1 (Vaccine coverage rates)	5472	7906	4856
Scenario 2.1 (Strain distribution 2014/15)	4665	6912	4124
Scenario 2.2 (Strain distribution 2015/16)	10,565	12,744	10,469
Scenario 2.3 (Strain distribution 2016/17)	1576	3357	612
Scenario 2.4 (Strain distribution 2017/18)	8254	9954	7253
Scenario 2.5 (Strain distribution 2018/19)	5713	9813	5294
Scenario 3 (aQIV rVE vs. SD-QIV at 13.9%)	18,183	23,326	19,055
Scenario 4.1 (lower bound SD-QIV VE)	668	1819	Dominant
Scenario 4.2 (upper bound SD-QIV VE)	12,470	17,005	12,755
Scenario 5.1 (lower bound rVEs)	41,221	51,276	44,790
Scenario 5.2 (upper bound rVEs)	386	1736	Dominant
Scenario 6.1 (30% decrease in complication costs)	6536	9123	6322
Scenario 6.2 (30% increase in complication costs)	4408	6688	3391
Scenario 7 (exclude HF)	7599	10,367	6,924

1 Euro = 10.18 NOK/ 7.44 DKK/ 10.68 SEK; aQIV: Adjuvanted quadrivalent influenza vaccine; HF: Heart failure; ICER: Incremental cost-effectiveness ratio; QALY: Quality adjusted life year; SD-QIV: Standard-dose quadrivalent influenza vaccine; rVE: Relative vaccine effectiveness; VE: Vaccine effectiveness.

Table S4. Scenario analysis results (aQIV vs. HD-QIV) – Healthcare payer perspective.

Scenario	NMB (€)		
	Denmark	Norway	Sweden
Base case	8,531,060	5,614,985	10,249,405
Scenario 1 (Vaccine coverage rates)	5,289,356	2,715,567	8,296,366
Scenario 2.1 (Strain distribution 2014/15)	8,421,393	5,551,396	10,058,734
Scenario 2.2 (Strain distribution 2015/16)	7,954,366	5,296,939	9,403,328
Scenario 2.3 (Strain distribution 2016/17)	8,886,945	5,825,479	10,740,354
Scenario 2.4 (Strain distribution 2017/18)	8,097,800	5,399,029	9,677,295
Scenario 2.5 (Strain distribution 2018/19)	8,309,844	5,405,009	9,898,486
Scenario 3 (aQIV rVE vs. SD-QIV at 13.9%)			
Scenario 4.1 (lower bound SD-QIV VE)	9,084,353	6,000,392	11,070,082
Scenario 4.2 (upper bound SD-QIV VE)	7,860,005	5,183,490	9,254,446

Scenario 5.1 (lower bound rVEs)	5,607,780 (Less effective)	3,780,702 (Less effective)	5,950,682 (Less effective)
Scenario 5.2 (upper bound rVEs)	10,213,302	6,677,630	12,716,258
Scenario 6.1 (30% decrease in complication costs)	8,289,451	5,464,037	9,870,187
Scenario 6.2 (30% increase in complication costs)	8,378,688	5,527,104	10,041,092
Scenario 7 (exclude HF)	8,152,054	5,382,759	9,703,936

1 Euro = 10.18 NOK/ 7.44 DKK/ 10.68 SEK; aQIV: Adjuvanted quadrivalent influenza vaccine; HF: Heart failure; NMB: Net monetary benefit; SD-QIV: Standard-dose quadrivalent influenza vaccine; rVE: Relative vaccine effectiveness; VE: Vaccine effectiveness.

Table S5. Scenario analysis results (aQIV vs. HD-QIV) - Societal perspective.

**Scenario**	**NMB (€)**		
	**Denmark**	**Norway**	**Sweden**
Base case	8,531,060	5,614,985	10,249,405
Scenario 1 (Vaccine coverage rates)	5,414,379	2,774,574	8,541,171
Scenario 2.1 (Strain distribution 2014/15)	8,630,568	5,678,318	10,367,103
Scenario 2.2 (Strain distribution 2015/16)	8,098,372	5,389,642	9,618,856
Scenario 2.3 (Strain distribution 2016/17)	9,161,083	5,989,259	11,145,276
Scenario 2.4 (Strain distribution 2017/18)	8,261,821	5,505,460	9,931,632
Scenario 2.5 (Strain distribution 2018/19)	8,503,454	5,512,245	10,184,155
Scenario 3 (aQIV rVE vs. SD-QIV at 13.9%)	NA	NA	NA
Scenario 4.1 (lower bound SD-QIV VE)	9,386,038	6,187,694	11,521,712
Scenario 4.2 (upper bound SD-QIV VE)	7,990,845	5,260,936	9,448,884
Scenario 5.1 (lower bound rVEs)	5,424,345 (Less effective)	3,669,505 (Less effective)	5,677,131 (Less effective)
Scenario 5.2 (upper bound rVEs)	10,672,519	6,956,005	13,401,074
Scenario 6.1 (30% decrease in complication costs)	8,486,442	5,583,452	10,163,952
Scenario 6.2 (30% increase in complication costs)	8,575,678	5,646,518	10,334,857
Scenario 7 (exclude HF)	8,325,347	5,487,739	9,962,027

1 Euro = 10.18 NOK/ 7.44 DKK/ 10.68 SEK; aQIV: Adjuvanted quadrivalent influenza vaccine; HF: Heart failure; NMB: Net monetary benefit; SD-QIV: Standard-dose quadrivalent influenza vaccine; rVE: Relative vaccine effectiveness; VE: Vaccine effectiveness.

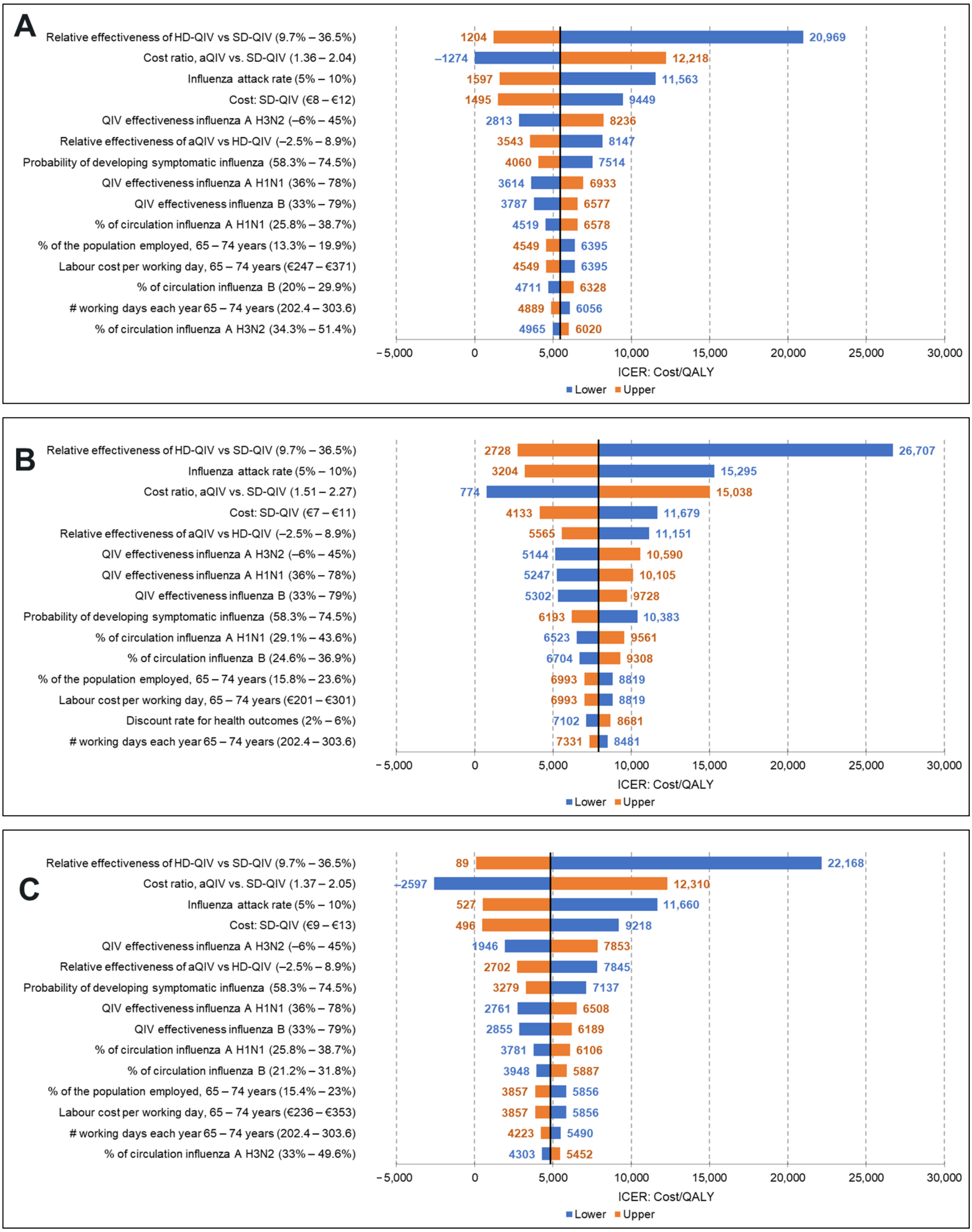

Figure S1. DSA results (aQIV vs. SD-QIV) – Societal perspective: (A) Denmark, (B) Norway, (C) Sweden.

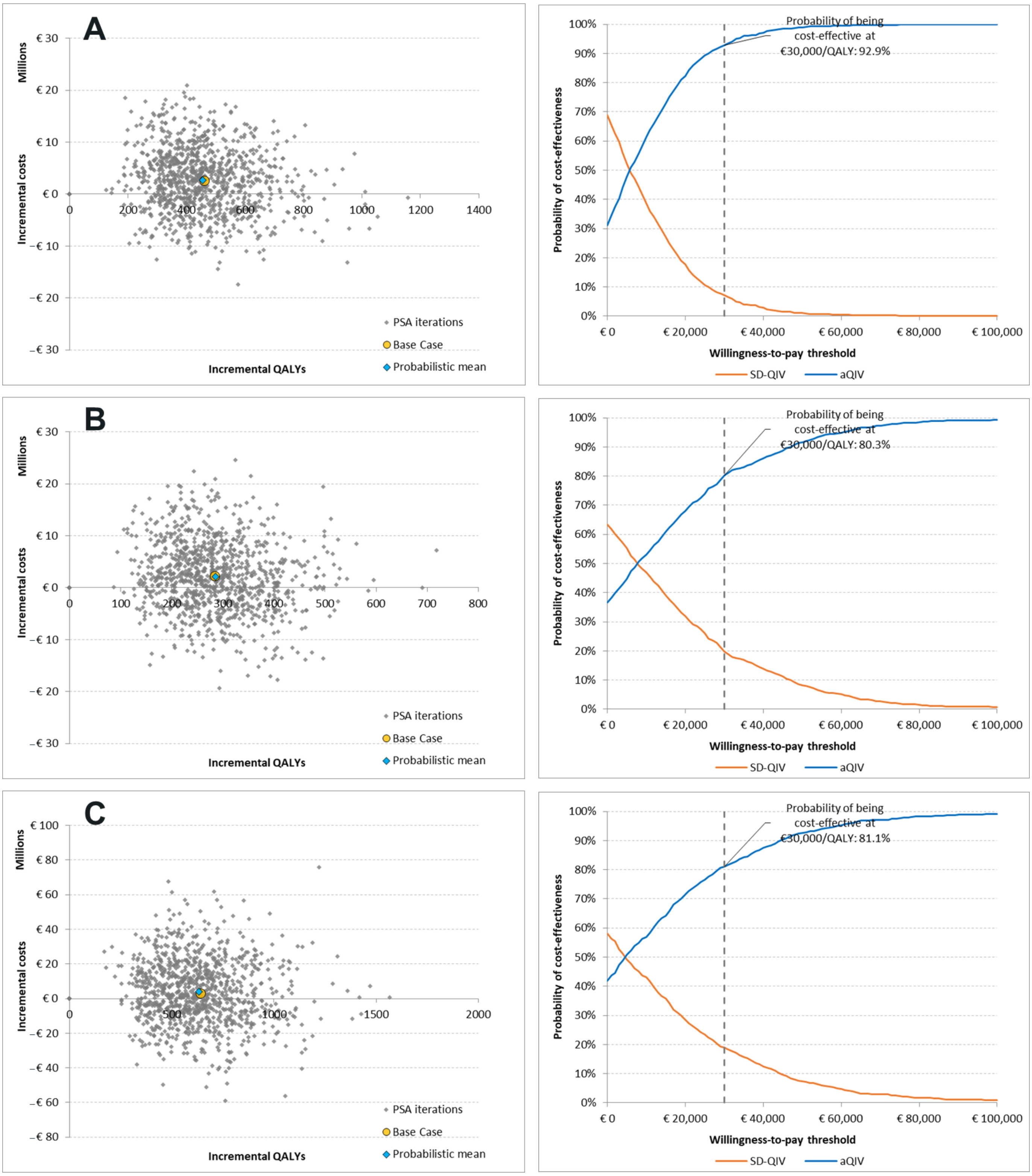

Figure S2. PSA results (aQIV vs. SD-QIV) – Societal perspective: (A) Denmark, (B) Norway, and (C) Sweden.

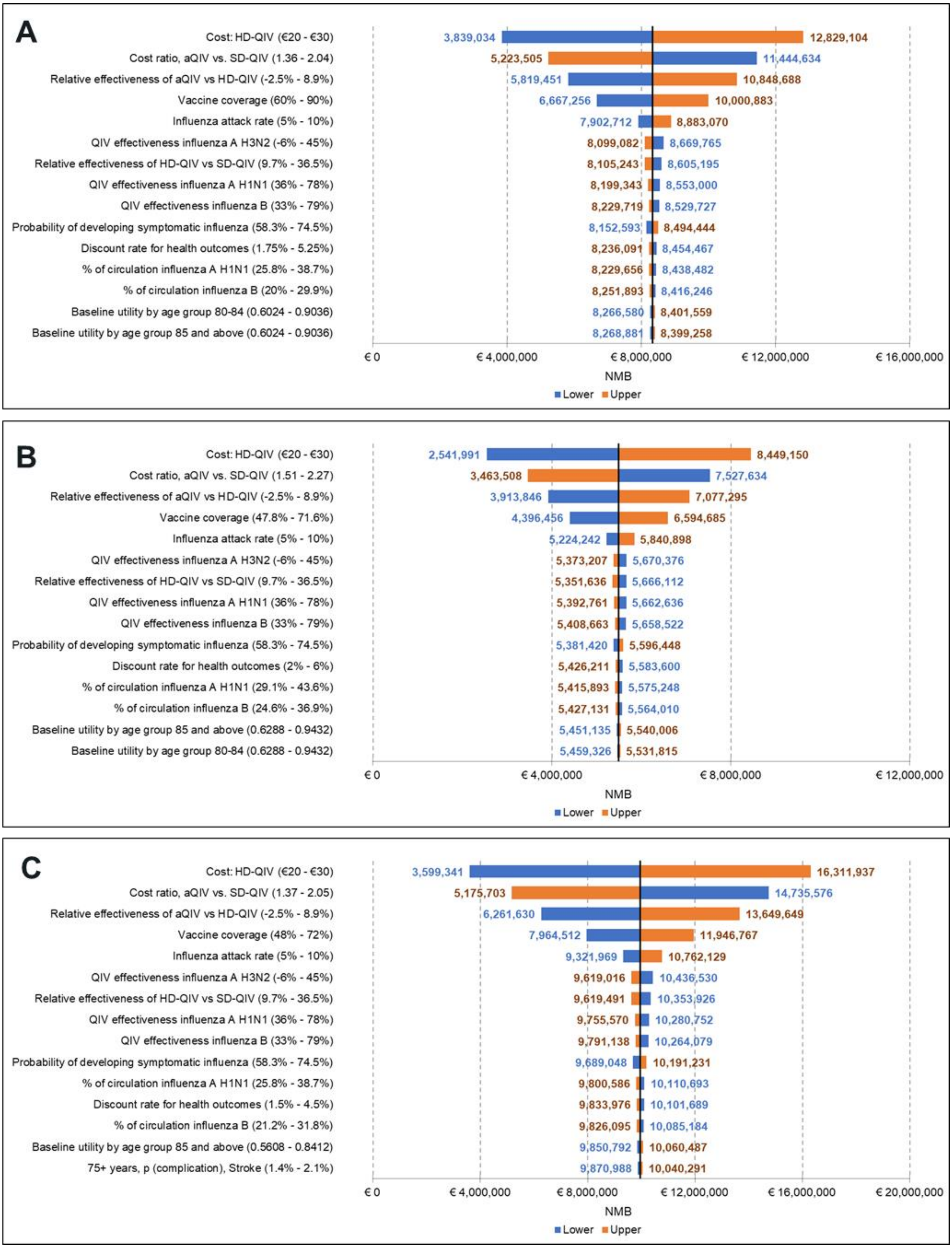

Figure S3. DSA results (aQIV vs. HD-QIV) – Healthcare payer perspective: (A) Denmark, (B) Norway, (C) Sweden.

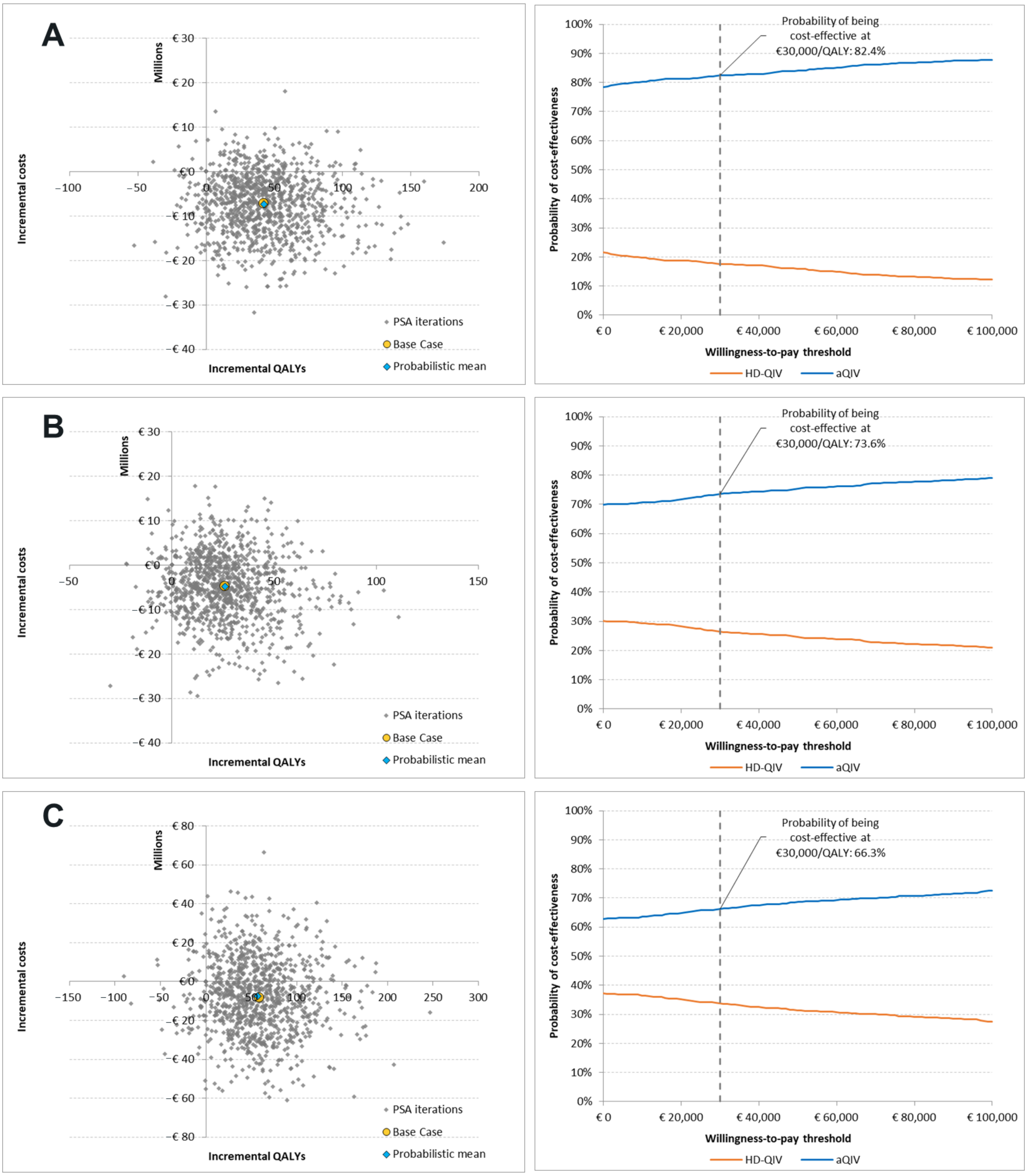

Figure S4. PSA results (aQIV vs. HD-QIV) – Healthcare payer perspective: (A) Denmark, (B) Norway, and (C) Sweden.

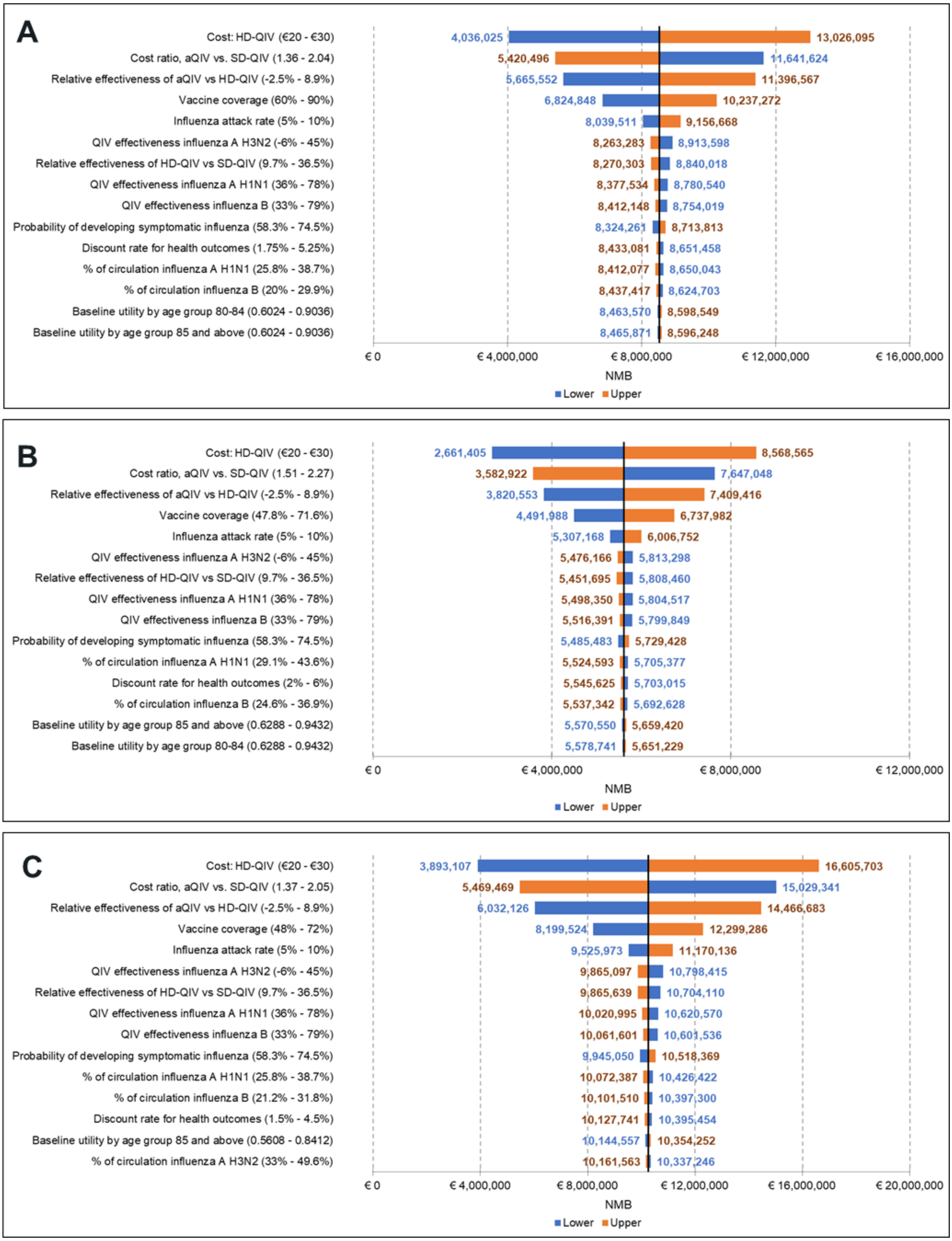

Figure S5. DSA results (aQIV vs. HD-QIV) – Societal perspective: (A) Denmark, (B) Norway, (C) Sweden.

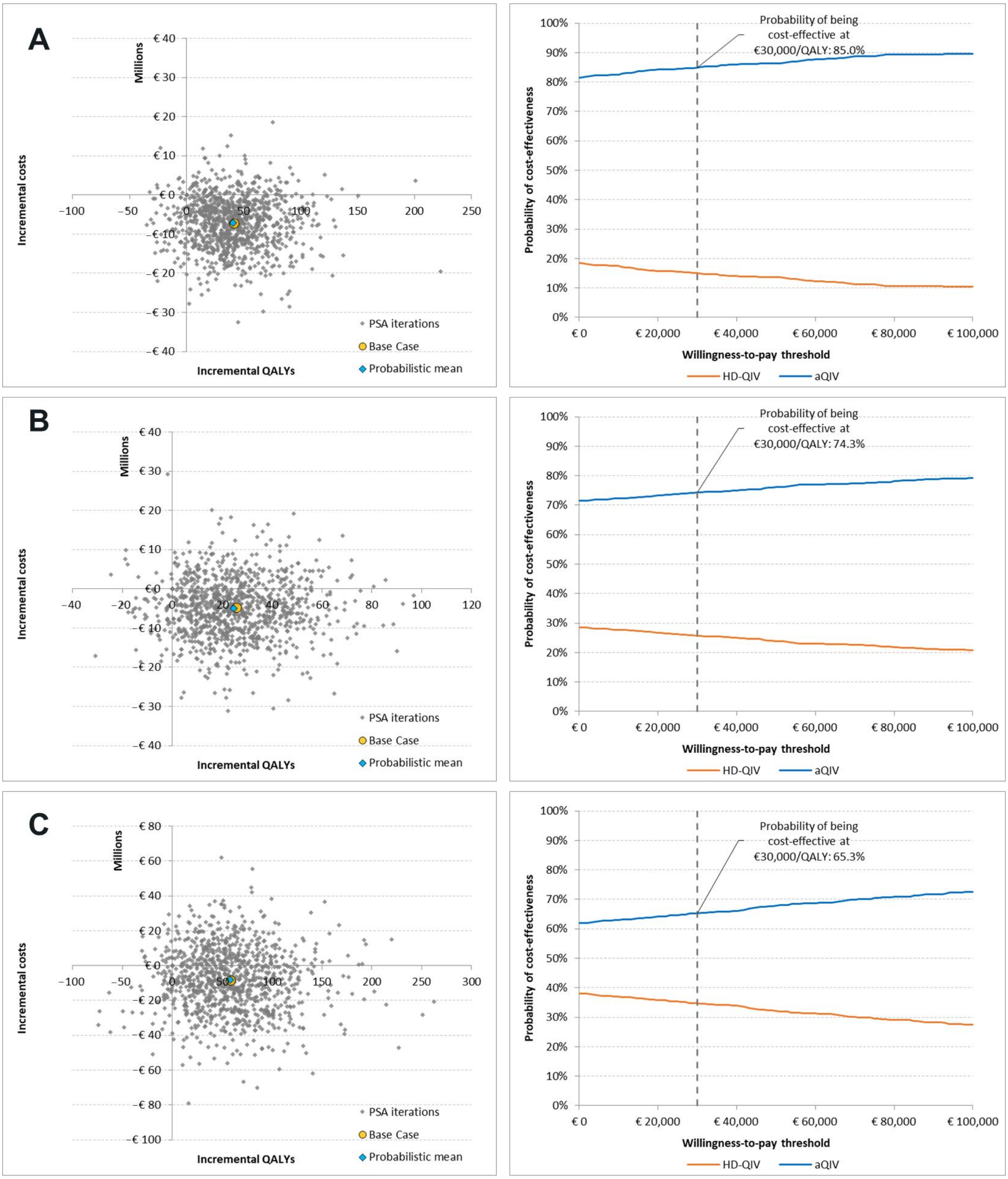

Figure S6. PSA results (aQIV vs. HD-QIV) – Societal perspective: (A) Denmark, (B) Norway, and (C) Sweden.

References

1. Cai, R.; Gerlier, L.; Eichner, M.; Schwehm, M.; Rajaram, S.; Mould-Quevedo, J.; Lamotte, M. Cost-effectiveness of the cell-based quadrivalent versus the standard egg-based quadrivalent influenza vaccine in Germany. *J Med Econ* **2021**, *24*, 490-501, doi:10.1080/13696998.2021.1908000.
2. Boehme, A.K.; Luna, J.; Kulick, E.R.; Kamel, H.; Elkind, M.S.V. Influenza-like illness as a trigger for ischemic stroke. *Ann Clin Transl Neurol* **2018**, *5*, 456-463, doi:10.1002/acn3.545.

3. Truelsen, T.; Piechowski-Jozwiak, B.; Bonita, R.; Mathers, C.; Bogousslavsky, J.; Boysen, G. Stroke incidence and prevalence in Europe: a review of available data. *Eur J Neurol* **2006**, *13*, 581-598, doi:10.1111/j.1468-1331.2006.01138.x.
4. Michaelidis, C.I.; Kern, M.S.; Smith, K.J. Cost-effectiveness of decision support strategies in acute bronchitis. *J Gen Intern Med* **2015**, *30*, 1505-1510, doi:10.1007/s11606-015-3289-3.
5. Rozenbaum, M.H.; Mangen, M.J.; Huijts, S.M.; van der Werf, T.S.; Postma, M.J. Incidence, direct costs and duration of hospitalization of patients hospitalized with community acquired pneumonia: A nationwide retrospective claims database analysis. *Vaccine* **2015**, *33*, 3193-3199, doi:10.1016/j.vaccine.2015.05.001.
6. Longstreth, G.F.; Feitelberg, S.P. Outpatient care of selected patients with acute non-variceal upper gastrointestinal haemorrhage. *Lancet* **1995**, *345*, 108-111, doi:10.1016/s0140-6736(95)90068-3.
7. Verpillat, P.; Dorey, J.; Guilhaume-Goulant, C.; Dabbous, F.; Brunet, J.; Aballea, S. A chart review of management of ischemic stroke patients in Germany. *J Mark Access Health Policy* **2015**, *3*, doi:10.3402/jmahp.v3.24223.
8. Fu, M.; Kontogeorgos, S.; Thunstrom, E.; Zverkova Sandstrom, T.; Kroon, C.; Bollano, E.; Schaufelberger, M.; Rosengren, A. Trends in myocarditis incidence, complications and mortality in Sweden from 2000 to 2014. *Sci Rep* **2022**, *12*, 1810, doi:10.1038/s41598-022-05951-z.
9. Engstad, T.; Engstad, T.; Viitanen, M.; Ellekjær, H. Epidemiology of stroke in the elderly in the Nordic countries. Incidence, survival, prevalence and risk factors. *Norsk Epidemiologi* **2012**, *22*, 121-126, doi:10.5324/nje.v22i2.1557.
10. Jones, N.R.; Roalfe, A.K.; Adoki, I.; Hobbs, F.R.; Taylor, C.J. Survival of patients with chronic heart failure in the community: a systematic review and meta-analysis. *European journal of heart failure* **2019**, *21*, 1306-1325.
11. Accordini, S.; Corsico, A.G.; Calciano, L.; Bono, R.; Cerveri, I.; Fois, A.; Pirina, P.; Tassinari, R.; Verlato, G.; de Marco, R. The impact of asthma, chronic bronchitis and allergic rhinitis on all-cause hospitalizations and limitations in daily activities: a population-based observational study. *BMC Pulm Med* **2015**, *15*, 10, doi:10.1186/s12890-015-0008-0.
12. Tellioglu, E.; Balci, G.; Mertoglu, A. Duration of stay of patients with community-acquired pneumonia in influenza season. *Turk Thorac J* **2018**, *19*, 182-186, doi:10.5152/TurkThoracJ.2018.17108.
13. Debes, S.; Haug, J.B.; de Blasio, B.F.; Jonassen, C.M.; Dudman, S.G. Etiology of viral respiratory tract infections in hospitalized adults, and evidence of the high frequency of prehospitalization antibiotic treatment in Norway. *Health Sci Rep* **2021**, *4*, e403, doi:10.1002/hsr2.403.
14. Kotseva, K.; Gerlier, L.; Sidelnikov, E.; Kutikova, L.; Lamotte, M.; Amarenco, P.; Annemans, L. Patient and caregiver productivity loss and indirect costs associated with cardiovascular events in Europe. *Eur J Prev Cardiol* **2019**, *26*, 1150-1157, doi:10.1177/2047487319834770.
15. Turchetti, G.; Bellelli, S.; Amato, M.; Bianchi, S.; Conti, P.; Cupisti, A.; Panichi, V.; Rosati, A.; Pizzarelli, F.; On Behalf of the Tuscany, C.K.D.S.G. The social cost of chronic kidney disease in Italy. *Eur J Health Econ* **2017**, *18*, 847-858, doi:10.1007/s10198-016-0830-1.
16. Strzelczyk, A.; Knake, S.; Oertel, W.H.; Rosenow, F.; Hamer, H.M. Inpatient treatment costs of status epilepticus in adults in Germany. *Seizure* **2013**, *22*, 882-885, doi:10.1016/j.seizure.2013.08.003.
17. Button, L.A.; Roberts, S.E.; Evans, P.A.; Goldacre, M.J.; Akbari, A.; Dsilva, R.; Macey, S.; Williams, J.G. Hospitalized incidence and case fatality for upper gastrointestinal bleeding from 1999 to 2007: a record linkage study. *Aliment Pharmacol Ther* **2011**, *33*, 64-76, doi:10.1111/j.1365-2036.2010.04495.x.
18. Stendardo, M.; Bonci, M.; Casillo, V.; Miglio, R.; Giovannini, G.; Nardini, M.; Campo, G.; Fucili, A.; Boschetto, P. Predicting return to work after acute myocardial infarction: Socio-occupational factors overcome clinical conditions. *PLoS One* **2018**, *13*, e0208842, doi:10.1371/journal.pone.0208842.
19. The Danish Health Data Authority. DRG rates 2022. Available online: https://sundhedsdatastyrelsen.dk/da/afregning-og-finansiering/takster-drg/takster-2022 (accessed on 31 October 2022).
20. The Norwegian Directorate of Health. Effort-driven financing (ISF) – Regulations 2022. Available online: https://www.helsedirektoratet.no/tema/finansiering/innsatsstyrt-finansiering-og-drg-systemet/innsatsstyrt-finansiering-isf (accessed on 31 October 2022).
21. Sweden Southern healthcare region. Regional prices and allowances. Available online: https://sodrasjukvardsregionen.se/download/regionala-priser-och-ersattningar-for-sodra-sjukvardsregionen-2022/?wpdmdl=24791&masterkey=61decc148fc78 (accessed on 15 November 2022).
22. The Danish Health Data Authority. DRG rates 2017: outpatient day rates Available online: https://sundhedsdatastyrelsen.dk/da/afregning-og-finansiering/takster-drg/takster-2017 (accessed on 31 October 2022).
